# Are ChatGPT, My AI Snapchat, and Metaverse used by dental students as reliable sources of dental education?

**DOI:** 10.3389/fdmed.2025.1673536

**Published:** 2026-01-06

**Authors:** Mohamed Ahmed Elsayed, Kusai Baroudi, Mohamed Anas Patni, Mahmoud Mohamed Elwakil, Nallan CSK Chaitanya, Mohmed Isaqali Karobari

**Affiliations:** 1 Department of Endodontics, RAK College of Dental Sciences, RAK Medical and Health Sciences University, Ras Al-Khaimah, United Arab Emirates; 2 Department of Endodontics, Faculty of Dentistry, Assiut University, Assiut, Egypt; 3 Department of Clinical Sciences, College of Dentistry, Ajman University, Ajman, United Arab Emirates; 4 Centre of Medical and Bio-allied Health Sciences Research, Ajman, United Arab Emirates; 5 Postgraduate Program in Health Sciences, University of Taubate, Taubaté, Brazil; 6 Department of Community Medicine, RAK College of Medicine Sciences, RAK Medical and Health Sciences University, Ras Al Khaimah, United Arab Emirates; 7 Department of Clinical Sciences, RAK College of Dental Sciences, RAK Medical and Health Sciences University, Ras Al-Khaimah, United Arab Emirates; 8 Department of Oral Radiology, RAK College of Dental Sciences, RAK Medical and Health Sciences University, Ras Al-Khaimah, United Arab Emirates; 9 Department of Conservative Dentistry and Endodontics, Saveetha Dental College and Hospitals, Saveetha Institute of Medical and Technical Sciences, Saveetha University, Chennai, Tamil Nadu, India

**Keywords:** ChatGPT, artificial intelligence, My AI Snapchat, Metaverse, dental students, dental education

## Abstract

A profound understanding of dental students' use of artificial intelligence (AI) applications and their perceptions is essential for promoting responsible adoption and guiding integration into dental education.

**Aim:**

This study evaluated dental students' perceptions, usage patterns, and trust in ChatGPT, Snapchat's My AI, and the Metaverse as educational tools.

**Materials and methods:**

A cross-sectional online survey was administered to undergraduate dental students at RAK College of Dental Sciences, United Arab Emirates, between February and May 2024. The questionnaire comprised 29 questions organized into five sections. The questions were formulated as multiple-choice questions. Descriptive statistics were calculated. Pearson's chi-square tests with *post-hoc* adjusted standardized residuals (ASRs) and Bonferroni corrections examined categorical associations. Ordinal logistic regression assessed predictors of AI awareness, and paired-samples t-tests with Cohen's d compared trust in ChatGPT vs. Snapchat AI.

**Results:**

The response rate was 57%, of which 70% were females. Self-rated awareness of AI increased across academic years, with fifth-year students rating their awareness score as 4 ± 0.9 out of 5, compared to 3 ± 1.4 in the first year. Ordinal regression showed no significant effects of year, gender, or their interaction (*p* > 0.19). ChatGPT was the most used tool (81.5%), followed by grammar correction tools (75%) and Snapchat AI (74.4%), while Metaverse use was limited (28.6%). Chi-square analyses confirmed significantly greater use of ChatGPT, Grammarly, and Snapchat AI compared with Metaverse (*p* < 0.001). Fourth-year students most often used AI for academic or clinical purposes (41.7%). Educational potential was endorsed by 78% of students, while privacy and data security were the predominant concerns (78%). Compared with Snapchat AI, ChatGPT was significantly more often used for education, preferred for quick responses, and more frequently associated with positive beliefs about future learning. Paired-samples t-tests demonstrated consistently higher trust in ChatGPT across all academic years, with moderate-to-large effect sizes (Cohen's d = 0.67–0.91; *p* < 0.01).

**Conclusions:**

Dental students reported widespread adoption and higher trust in ChatGPT compared to other AI tools. While recognizing its educational potential, concerns about accuracy and privacy underscore the need for integrating AI literacy and evidence-based evaluation into dental curricula.

## Introduction

Artificial intelligence (AI) has rapidly expanded across healthcare and education, offering tools that enhance information retrieval, problem-solving, and decision support (1). AI is a transdisciplinary field that involves the use of computer algorithms to model intelligent behaviors with minimal human intervention. AI applications are primarily based on machine learning and utilize information retrieval, image recognition, and cognitive decision-support systems (2). In dentistry, AI has demonstrated applications in radiographic interpretation, diagnostic support, treatment planning, and simulation-based learning (3). Beyond clinical practice, AI has the potential to transform dental education by providing students with interactive platforms, immediate feedback, and supplementary learning resources that complement traditional curricula (4).

Among available AI applications, ChatGPT (OpenAI, San Francisco, USA) has gained significant attention for its ability to generate coherent, human-like responses and assist with academic tasks. Several studies have shown that dental students and educators are increasingly using ChatGPT for literature support, assignment preparation, and clarification of complex concepts (5–7). While its accessibility and versatility are attractive, concerns persist regarding the accuracy and reliability of its outputs, particularly in healthcare education, where evidence-based information is critical (8). Similarly, Snapchat's My AI chatbot, powered by the same underlying language model, has become widely available to students as a mobile application. Although designed for general use, its easy accessibility raises questions about whether students employ it for academic support and how it compares with ChatGPT in terms of trust and perceived utility (9). In addition, the Metaverse, which integrates virtual and augmented reality, has been increasingly explored in medical and dental training as an immersive tool for clinical simulation and skill acquisition (10). The application of the metaverse in education still faces challenges such as cost, accessibility, privacy, and data security (11).

Despite the potential advantages of these AI applications in dental education, these tools may lack the depth, accuracy, and contextual relevance of information provided by trusted sources such as textbooks and evidence-based educational materials (12). While prior studies have examined dental students' awareness and attitudes toward AI (13, 14), most have focused on general perceptions rather than specific applications currently available to students.

Understanding the current usage patterns of these applications among dental students, as well as the extent of their frequency of use and their level of awareness of AI tools, will greatly assist in incorporating these applications into dental curricula in the future. The present study aims to evaluate the perceptions, usage patterns, and trust of dental students in relation to ChatGPT, My AI Snapchat, and the Metaverse. Specifically, it investigates the frequency of use of these applications for academic purposes, the types of information sought, and students' subjective assessment of the accuracy and reliability of the information received. The findings may inform strategies to integrate AI tools into dental education in a manner that enhances learning while upholding academic rigor and reliability.

## Methods

A comprehensive questionnaire was developed in the English language following a thorough analysis of questions used in previous studies with similar objectives (15). The formulation process adhered to specific guidelines for survey reporting (16) and was overseen by experts in the field. The study was conducted in accordance with the Declaration of Helsinki, and the proposal was approved by the Institutional Review Board (RAKCODS-REC−18−2023/24-UG). The self-administered questionnaire was hosted on Google Forms and distributed along with a cover letter clearly outlining the study's objectives and the measures taken to safeguard participant anonymity. To ensure the clarity, validity, and effectiveness of the questionnaire, it underwent rigorous evaluation by two dental professors. Participants were assured of their anonymity in the survey, and the invitation explicitly stated that there were no incentives or penalties associated with participation in the study. Before the main survey was conducted, a pilot test was carried out on a small group of students to assess the clarity, feasibility, and applicability of the questionnaire. The link to the Google Form was emailed to all dental students twice between February and May 2024.

### Questionnaire structure

The Google Forms questionnaire comprised 29 questions organized into five sections. Questions are formulated as multiple-choice questions. The introduction clarifies the research title and aims of the study. A mandatory informed consent was introduced: “Do you agree to participate in this study?” with “yes” as the answer being required to move into the next section of the survey, while the answer of “no” resulted in the closure of the survey. The first section was demographic information and included two questions, which were gender and the grade of dental education. In the second section, the participants were asked six questions about their awareness and experience of using various AI tools. The third section comprised 9 questions regarding the perceived benefits and challenges of AI in dental education. The fourth section was related to the specific use and experience of ChatGPT and included six questions. The fifth section was specific to the use and experience of Snapchat's My AI feature and included six questions (Supplementary Appendix S1).

### Statistical and data analysis

Data were analyzed using IBM SPSS Statistics, version 29 (IBM Corp., Armonk, NY, USA). Descriptive statistics were computed for all variables and are presented as frequencies and percentages for categorical data and as mean ± standard deviation (SD) or median with interquartile range (IQR) for continuous and ordinal data, as appropriate. The required sample size was calculated using a 95% confidence level, 5% margin of error, estimated population proportion of 50%, and a total population size of 295 students, yielding a minimum target of 167 participants.

Associations between categorical variables, such as AI tool usage status, frequency of use, and reasons for preference, and beliefs about educational potential, were examined using Pearson's chi-square tests. Contingency tables were constructed for comparisons, including (i) AI tool (ChatGPT, Snapchat AI, Metaverse, Grammarly) × usage status (ever vs. never used), (ii) AI tool × reason for preference (quick responses, trustable answers, not used), and (iii) software type (ChatGPT vs. Snapchat AI) × beliefs about enhancing dental education (strongly disagree to strongly agree). For tables yielding significant omnibus chi-square results, *post-hoc* analyses were performed using adjusted standardized residuals (ASRs). Bonferroni corrections were applied to control for multiple comparisons, with adjusted thresholds (e.g., ±2.73 for 2 × 4 tables and ±2.81 for 2 × 5 tables) used to determine significance.

Paired-samples t-tests were performed within each academic year to directly compare trust ratings between ChatGPT and Snapchat AI. For comparing paired trust scores between ChatGPT and Snapchat AI, both parametric and non-parametric tests were applied. Normality of within-subject differences (ChatGPT—Snapchat AI) was verified using Q–Q plots and the Shapiro–Wilk test. As the distribution deviated from normality (*p* < 0.05), a Wilcoxon signed-rank test was additionally performed as a sensitivity analysis to confirm the robustness of results. The paired-samples t-test was retained as the primary analysis, given the sufficiently large sample size and the robustness of the t-test to moderate deviations from normality.

Effect sizes were calculated using Cohen's *d*, along with 95% confidence intervals (CIs). Since five paired t-tests were conducted to compare ChatGPT and Snapchat AI trust scores across academic years (Year 1 to Year 5), each set of yearly comparisons was considered a single family of related tests. To control for type I error inflation due to multiple testing, a Bonferroni correction was applied. The adjusted significance threshold for these comparisons was set at *α* = 0.05/5 = 0.01. Within each family, *p*-values below 0.01 were considered statistically significant. To further investigate factors influencing students' trust and awareness of AI-based software tools, a series of ordinal logistic regression models using the cumulative logit link function were employed. Separate models were developed for each dependent variable: trust in ChatGPT, trust in Snapchat AI, and general AI awareness. The predictor variables included year of study, gender, and their interaction term (Year × Gender) to examine potential combined effects. Model fit was evaluated using the −2 Log Likelihood ratio test, along with Pearson and Deviance *χ*^2^ statistics to assess overall model adequacy. The Test of Parallel Lines was applied to confirm the proportional odds assumption. Nagelkerke R^2^ was reported as a measure of the variance explained by the model. Results included *β* coefficients (log-odds), Wald *χ*^2^ statistics, *p*-values, and Adjusted Odds Ratios (AOR = *e*^*β*) with corresponding 95% confidence intervals.

## Results

**Figure 1:**
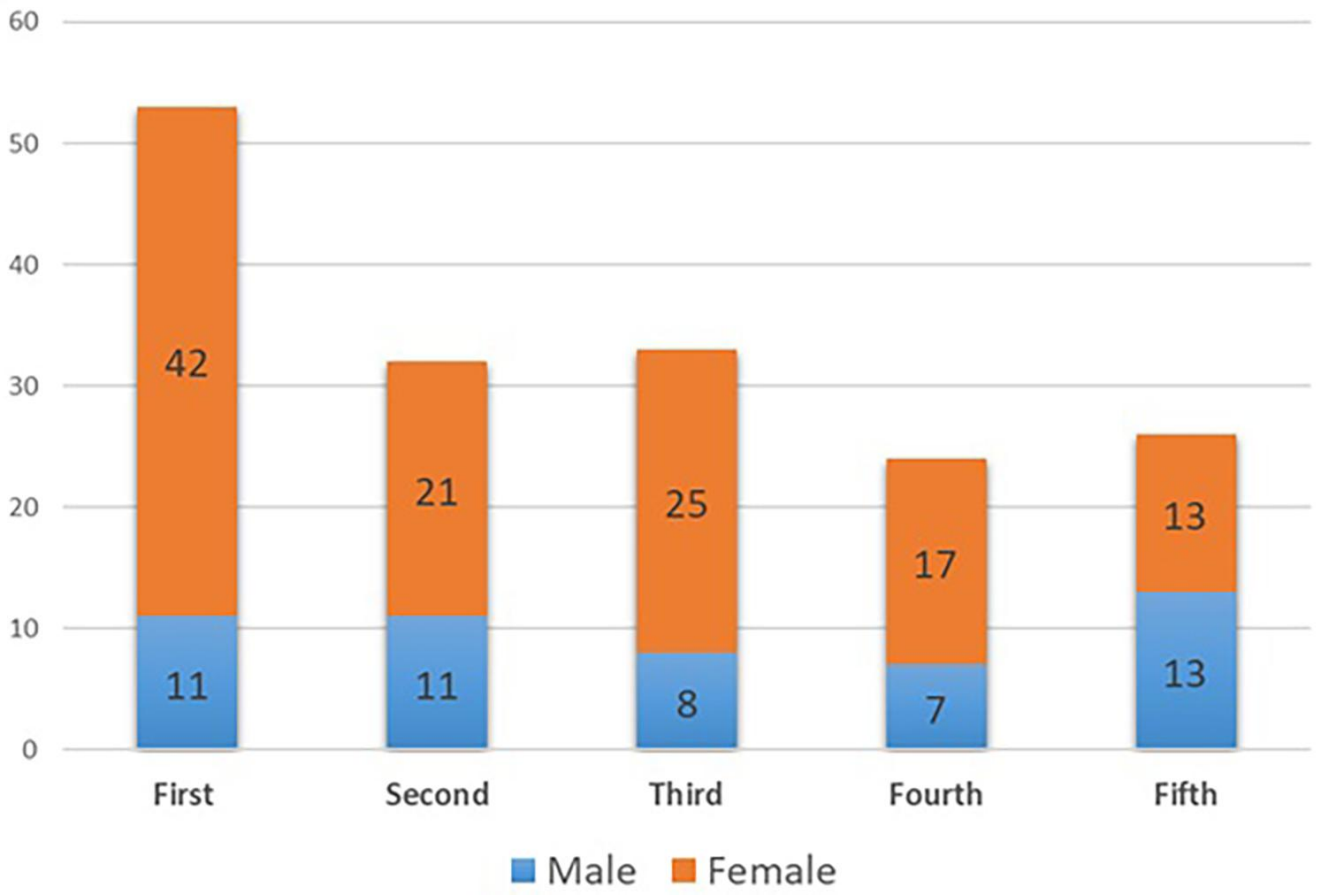
Distribution of participants based on year of study and gender.

**Figure 2:**
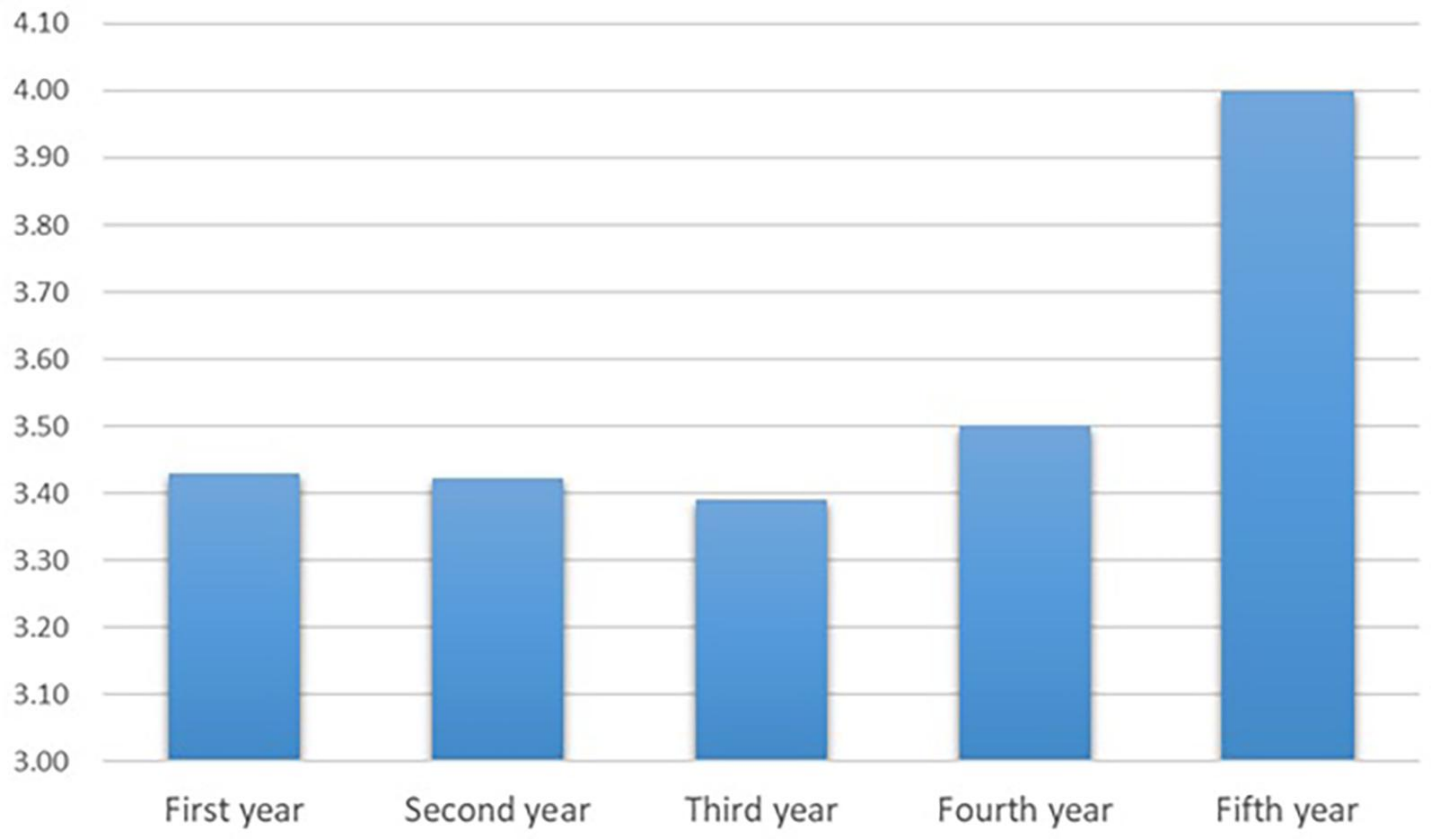
Self-rated awareness of artificial intelligence (AI) applications among dental students. Awareness was assessed using a 5-point Likert scale (1 = very low; 5 = very high).

Out of 295 students invited to participate in the study, 168 responded, yielding a response rate of 57%. Among the respondents, 70% were females (*n* = 118) and 30% were males (*n* = 50). The distribution of participants across academic years showed that first-year students comprised the largest group at 32% (*n* = 53), followed by third-year students at 20% (*n* = 33), second-year students at 19% (*n* = 32), fifth-year students at 15% (*n* = 26), and fourth-year students at 14% (*n* = 24) (Figure 1). Analysis of self-rated awareness of AI applications measured on a 5-point Likert scale showed a progressive increase across academic years, with fifth-year students reporting the highest median score (4, IQR = 3–5) compared to first-year students (3, IQR = 2–5) (Figure 2). Mann–Whitney U tests revealed no significant gender differences in awareness either within individual years or overall (*U* = 3,164.0, *p* = 0.445) (Table 1). Ordinal logistic regression was performed with AI awareness as the dependent variable and year of study, gender, and their interaction as predictors. None of the interaction terms between year and gender reached statistical significance (all *p*-values >0.19). For instance, awareness levels among fifth-year females (*β* = 1.046, 95% CI: −0.543 to 2.635, *p* = 0.197) and fifth-year males (*β* = 0.961, 95% CI: −0.624 to 2.546, *p* = 0.235) did not significantly differ from the reference group (third-year males). The overall model was not statistically significant, *χ*^2^(9) = 9.34, *p* = 0.41, and accounted for only a small proportion of the variance (*N*agelkerke *R*^2^ = 0.057) (Table 2). These results suggest that year of study, gender, and their interaction did not significantly predict dental students' awareness of AI applications.

**Table 1:** Mean ± standard deviation (SD) scores of dental students’ self-rated awareness of artificial intelligence (AI) applications on a 5-point Likert scale (1 = very low; 5 = very high).

Year of Study	Total	Male	Females	Mann–Whitney *U*	*p*-value
	Mean (SD)	Mean (SD)	Mean (SD)		
	Median(IQR)	Median(IQR)	Median(IQR)		
1st year	3.33 (1.41)	3.27 (1.61)	3.35 (1.37)	228.0	0.946
	3 (2–5)	3 (2–4)	4 (2–4.25)		
2nd year	3.09 (1.49	3.46 (1.80)	2.89 (1.3)	144.0	0.271
	3 (2–4.25)	4 (2–5)	3 (2–4)		
3rd year	3.36 (1.19)	3.12 (1.55)	3.44 (1.08)	94.0	0.821
	3 (3–4)	3.5 (2.25–4)	3 (2.5–4)		
4th year	3.54 (1.14)	3.43 (1.27)	3.59 (1.12)	53.5	0.693
	3.5 (3–4.25)	3 (2–5)	4 (3–4.5)		
5th year	4.01 (0.89)	4.00 (0.81)	4.00 (1)	82.0	0.92
	4 (3–5)	4 (3–5)	4(3–5)		

Overall comparison: Male vs. Female: U=3,164.0, *p*=0.445.

**Table 2:** Ordinal logistic regression predicting AI awareness by year of study, gender, and their interaction.

Predictor	Estimate (*β*)	Std. error	Wald *χ*^2^	df	*p*-value	95% CI (Lower, Upper)	AOR (e^β)
Year = Fifth × Female	1.046	0.811	1.665	1	0.197	−0.543, 2.635	2.85
Year = Fifth × Male	0.961	0.809	1.412	1	0.235	−0.624, 2.546	2.61
Year = First × Female	0.203	0.687	0.088	1	0.767	−1.144, 1.551	1.23
Year = First × Male	0.110	0.828	0.018	1	0.894	−1.513, 1.732	1.12
Year = Fourth × Female	0.452	0.765	0.349	1	0.554	−1.047, 1.952	1.57
Year = Fourth × Male	0.169	0.922	0.034	1	0.854	−1.638, 1.977	1.18
Year = Second × Female	−0.497	0.741	0.449	1	0.503	−1.950, 0.956	0.61
Year = Second × Male	0.617	0.831	0.551	1	0.458	−1.012, 2.246	1.85
Year = Third × Female	0.194	0.724	0.072	1	0.789	−1.225, 1.613	1.21

Reference group: Year = Third, Gender = Male. Model fit: *χ*^2^(9) = 9.34, *p* = 0.41. Nagelkerke *R*^2^ = 0.057.

**Figure 3:**
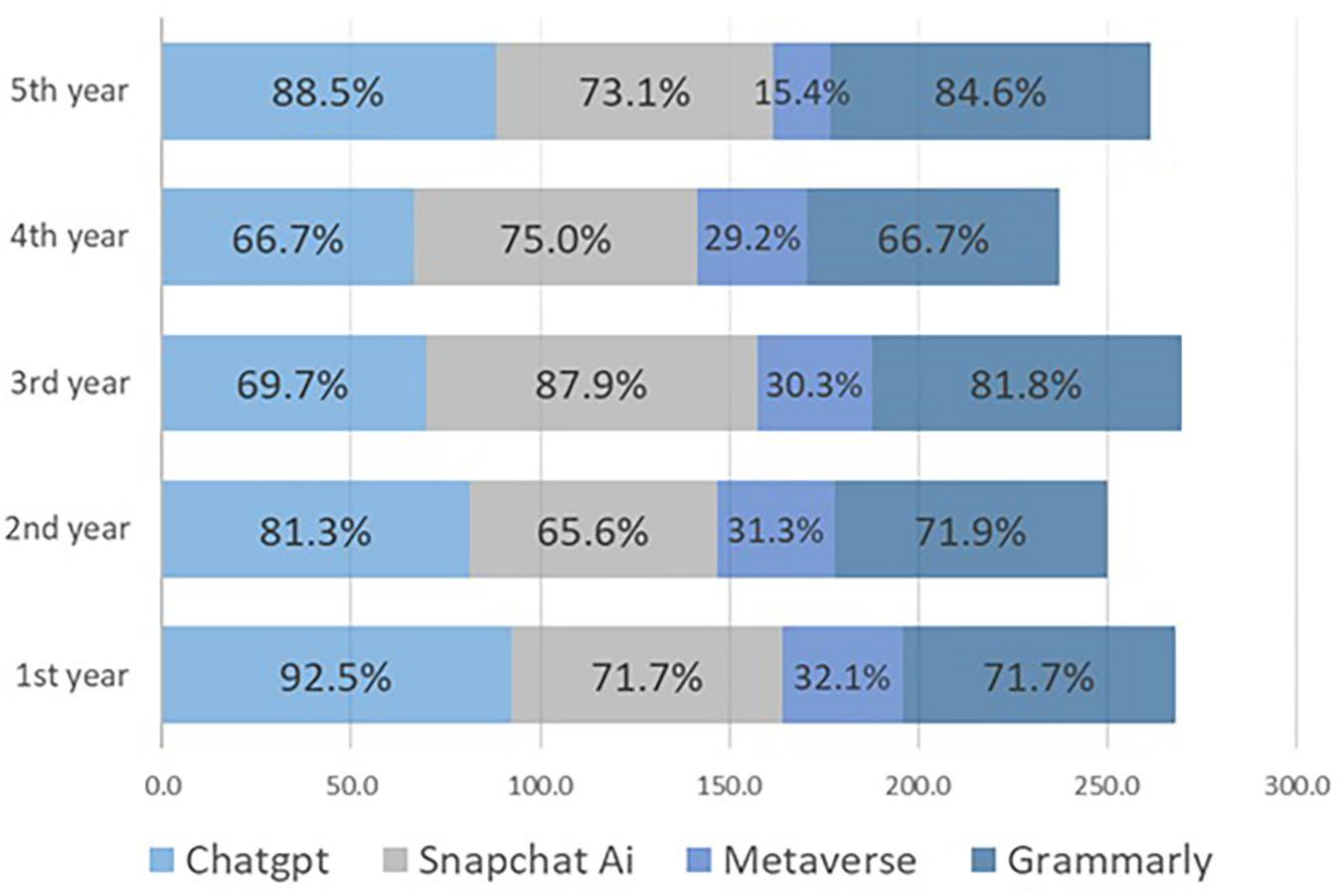
Academic year-wise use of AI applications by dental students. Proportions of students reporting ever or never using ChatGPT, Snapchat's My AI, the Metaverse, or grammar correction tools.

With respect to AI tool usage, ChatGPT was the most frequently used application (81.5%), followed by grammar correction tools (75%) and Snapchat AI (74.4%). In contrast, only 28.6% had experience with the Metaverse. Significant differences were observed in reported usage of AI applications (*χ*^2^ = 131.9, df = 3, *p* < 0.0001). *post-hoc* residual analysis with Bonferroni correction (*α* = 0.00625; threshold ±2.73) revealed that ChatGPT, Grammarly, and Snapchat AI were used significantly more often than expected, whereas Metaverse was used significantly less often (Table 3). Interestingly, first-year students had the highest percentage of metaverse usage (32.1%), while fifth-year students had the lowest (15.4%) (Figure 3).

**Table 3:** Frequencies and percentages of dental students who reported ever using or never using four artificial intelligence (AI) applications (ChatGPT, snapchat AI, metaverse, and grammar correction tools).

Software	Ever used (%)	Never used (%)	*χ*^2^ value	*p*-value	ASR (ever used)	ASR (never used)
*ChatGPT*	137 (81.5)	31 (18.5)	131.9	<0.0001*	+5.2	−5.2
*Snapchat AI*	125 (74.4)	43 (25.6)			+3.0	−3.0
*Metaverse*	48 (28.6)	120 (71.4)			−11.4	+11.4
*Grammar correction tool*	126 (75)	42 (25)			+3.0	−3.0

ASR, Adjusted Standardized Residual. Bonferroni correction was applied (*α* = 0.05/8 = 0.00625, cutoff ±2.73). Cells with residuals exceeding ±2.73 are significant contributors and are shown in bold in the interpretation.

* Indicates that it is statistically significance difference.

When asked about the use of AI tools as resources for studies or dental practice, fourth-year students reported the highest utilization rate (41.7%), followed by second-year students (28.1%), with the lowest utilization seen among first-year students (17%). However, the chi-square test found no statistically significant association between AI usage and academic year (*χ*^2^ = 6.87, *p* = 0.143) (Table 4).

**Table 4:** Frequencies and percentages of dental students who reported using any AI tool as an information resource for academic studies or clinical dental practice, stratified by academic year.

Year of Study	Ever used	Never used	*χ*^2^ value	*p*-value
	*N* (%)	*N* (%)		
*1st year*	9 (17)	44 (83)	6.87	0.143
*2nd year*	9 (28.1)	43 (71.9)		
*3rd year*	6 (18.2)	27 (81.8)		
*4th year*	10 (41.7)	14 (58.3)		
*5th year*	5 (19.2)	21 (80.8)		

**Figure 4:**
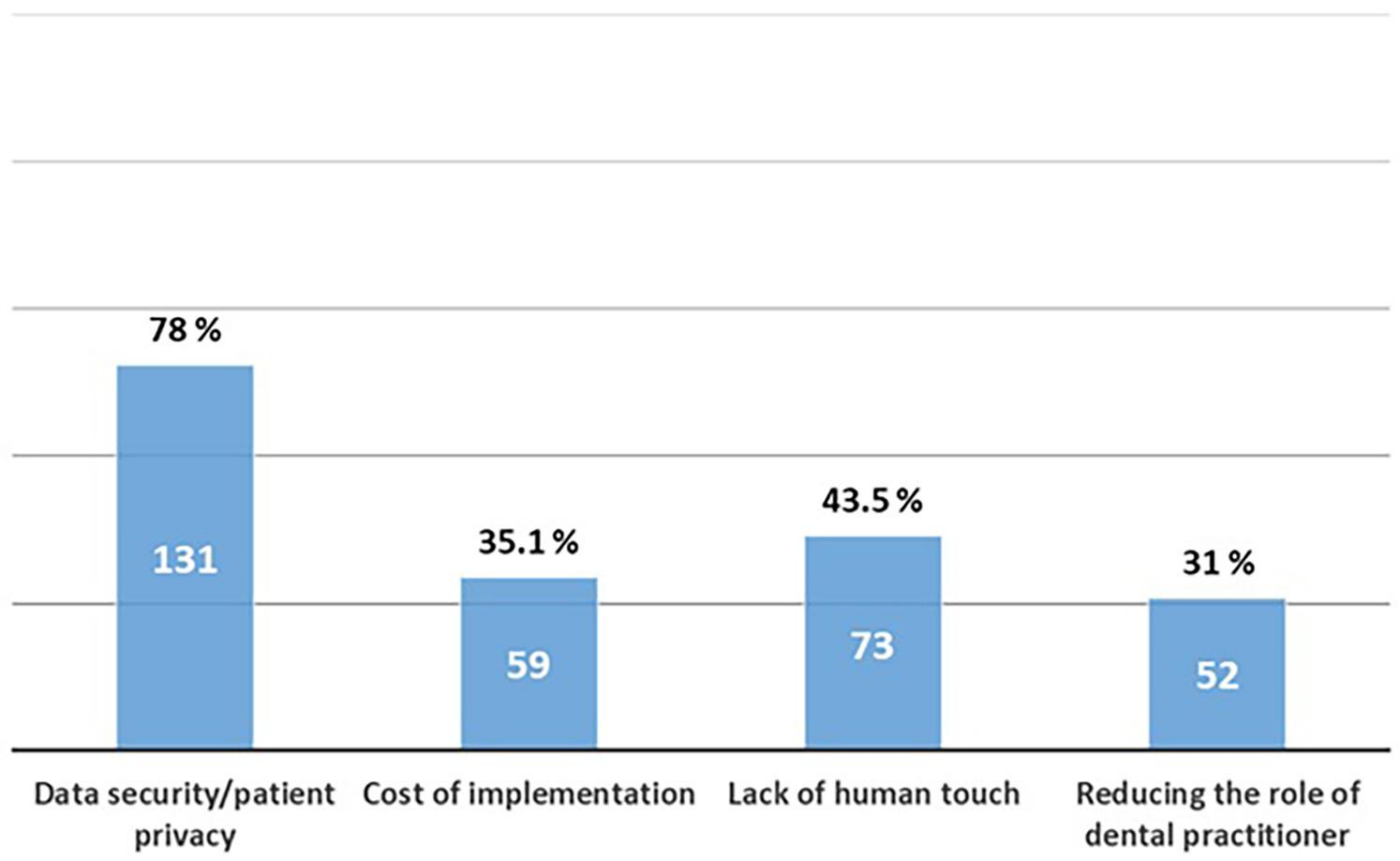
Perceived potential applications of AI in dentistry. Participants indicated the aspects of dentistry where they believed AI has the most potential.

**Figure 5:**
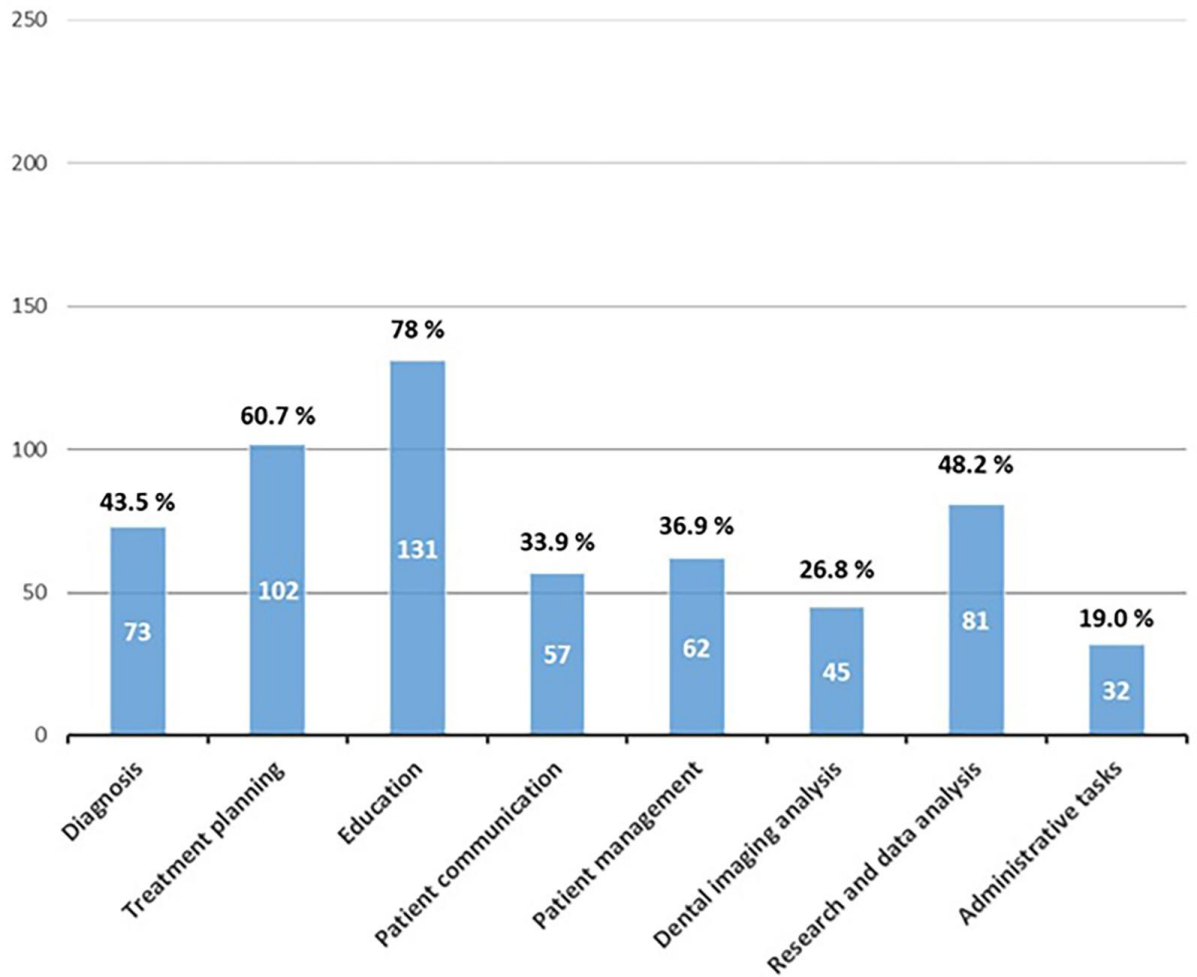
Concerns of dental students regarding the integration of AI into dentistry.

Regarding students' perceptions of AI applications in dentistry, 78% believed that AI has potential in dental education, 60.7% identified its usefulness in treatment planning, and 48% saw potential in research and data analysis. Conversely, administrative tasks were the least selected domain, with only 19% of students recognizing their potential (Figure 4). When addressing concerns about AI integration into dentistry, the most common issue was data security and patient privacy (78%), whereas only 31% expressed concern about AI reducing the role of dental practitioners in the future (Figure 5).

Comparisons between ChatGPT and Snapchat AI demonstrated notable differences. For educational use, ChatGPT was significantly more often reported as a source than Snapchat AI (*χ*^2^ = 21.7, df = 4, *p* < 0.001). *post-hoc* analysis with Bonferroni correction (*α* = 0.005, cutoff ±2.81) indicated that significantly fewer students reported “never” using ChatGPT for education, whereas significantly more students reported “never” using Snapchat AI. Regarding reasons for preference, a significant association was found between the main reason for preferring an AI tool and the type of software (*χ*^2^ = 21.61, df = 2, *p* < 0.001). *Post-hoc* analysis with Bonferroni correction (*α* = 0.0083, cutoff ±2.64) revealed that significantly more students (63.7%) reported ChatGPT as providing “quick and effortless responses” (ASR > +2.7), whereas significantly more students reported Snapchat AI as “not used as a source of information” (ASR > +2.8). No significant difference was observed for “trustable answers”.

Students' perceptions of privacy concerns did not differ significantly between ChatGPT and Snapchat AI (*χ*^2^ = 6.58, *p* = 0.08). However, beliefs about the potential of these tools to enhance future learning showed marked differences: 50.6% of participants agreed or strongly agreed that ChatGPT could positively impact dental education, compared with only 16.7% for Snapchat AI. A significant association was found between the type of software and belief about enhancing learning in dental education (*χ*^2^ = 50.39, *p* < 0.0001). *post-hoc* analysis with Bonferroni correction (*α* = 0.005, cutoff ±2.81) indicated that ChatGPT was significantly more often associated with “Agree,” while Snapchat AI was more often associated with “Neutral” and “Strongly disagree” responses (Table 5). Trust scores for ChatGPT were consistently higher across all academic years compared to Snapchat AI. Paired-samples *t*-tests revealed that, across all academic years, students consistently reported significantly greater trust in ChatGPT compared to Snapchat AI (all *p* < 0.001, except for fourth-year students, *p* = 0.003). The observed effect sizes ranged from moderate to large (Cohen's *d* = 0.67–0.91). After applying Bonferroni correction (*α* = 0.01 for five paired comparisons), all results for trust differences between ChatGPT and Snapchat AI remained statistically significant (Table 6).

**Table 5:** Frequencies and percentages of dental students’ specific use and attitudes regarding ChatGPT and snapchat AI, including frequency of use, educational applications, reasons for preference, privacy concerns, and beliefs about future impact on dental education.

Dental students' specific use and attitudes	ChatGPT	Snapchat AI	*χ*^2^ value	*p*-value	ASR (ChatGPT)	ASR (Snapchat)
A. Frequency of use (%)						
Multiple times in a day	11 (6.5)	13 (7.7)	6.84	0.14	—	
Daily	17 (10.1)	12 (7.1)				
A few times a week	42 (25)	40 (23.8)				
Rarely	76 (45.2)	64 (38.1)				
Never	22 (13.1)	39 (23.2)				
B. Use of the tool as a source of education or dental information (%)						
All the time	14 (8.3)	14 (8.3)	21.7	<0.001*	0.0	0.0
Multiple times	36 (21.4)	24 (14.3)			1.7	−1.7
Few times	51 (30.4)	37 (22.0)			1.7	−1.7
Rarely	41 (24.4)	30 (17.9)			−4.6	+4.6
Never	26 (15.5)	63 (37.5)			1.5	−1.5
C. The main reason for preferring this software as a source of information(%)						
Quick and effortless responses	107 (63.7)	64 (38.1)	21.6	<0.0001*	+3.5	−3.5
Trustable answers	39 (23.2)	34 (20.2)			+0.7	−0.7
Not used as a source of information	22 (13.1)	65 (38.7)			−4.6	+4.6
D. Concerns about privacy or data security when using this tool						
Yes	64 (38.1)	57 (33.9)	6.58	0.08	—	
No	51 (30.4)	41 (24.4)				
May be	31 (18.4)	51 (30.4)				
Inapplicable	22 (13.1)	19 (11.3)				
E. Your belief regarding software's use can enhance overall learning in dental school in the future.						
Strongly agree	16 (9.5)	6 (3.6)	50.39	<0.0001*	+2.2	−2.2
Agree	69 (41.1)	22 (13.1)			+5.8	−5.8
Neutral	45 (26.7)	73 (43.5)			−3.2	+3.2
Disagree	32 (19.1)	39 (23.2)			−0.9	+0.9
Strongly disagree	6 (3.6)	28 (16.7)			−4.0	+4.0

ASR, Adjusted Standardized Residual. Bonferroni correction applied: cutoff ±2.81 for 5 × 2 tables (Sections B, E), cutoff ±2.64 for 3 × 2 table (Section C). ASR values were only reported for significant chi-square tests.

* Indicates that it is statistically significance difference.

**Table 6:** Paired samples t-test comparing mean (±SD) trust scores for ChatGPT and snapchat AI among dental students across years of study, measured on a 5-point Likert scale (1 = very low; 5 = very high).

Year of Study	Trust in ChatGPT (Mean ± SD)	Trust in Snapchat AI (Mean ± SD)	*t* value	*p*-value	Cohen's d (95%CI)
1st year	3.11 ± 1.12	1.72 ± 1.53	6.618	<0.0001	0.91 (0.59–1.23)
2nd year	2.97 ± 1.03	1.81 ± 1.55	4.016	<0.0001	0.71 (0.32–1.09)
3rd year	2.73 ± 0.94	1.82 ± 1.53	3.855	<0.0001	0.67 (0.29–1.05)
4th year	2.92 ± 1.10	1.92 ± 1.44	3.323	0.003	0.68 (0.23–1.12)
5th year	3.42 ± 0.80	2.26 ± 1.28	4.186	<0.0001	0.82 (0.37–1.26)
*p* value	0.124	0.65			

An ordinal logistic regression was performed to assess the effects of year of study, gender, and their interaction on levels of trust in ChatGPT. The overall model was statistically significant, *χ*^2^(9) = 21.78, *p* = 0.010, indicating that the inclusion of predictors significantly improved model fit. Goodness-of-fit indicators were acceptable, with Pearson *χ*^2^(27) = 21.43, *p* = 0.77 and Deviance *χ*^2^(27) = 25.86, *p* = 0.53, suggesting adequate model representation. The proportional-odds assumption was met (*p* = 0.357). The Nagelkerke R^2^ was 0.13, indicating modest explanatory power. Among predictors, only the interaction between being a fourth-year and male student was statistically significant (*β* = −2.13, *p* = 0.028, AOR = 0.12, 95% CI: 0.02–0.79), suggesting that fourth-year male students were significantly less likely to report higher levels of trust in ChatGPT compared with third-year male students. No other main effects or interactions reached statistical significance (Table 7).

**Table 7:** Ordinal logistic regression predicting trust in ChatGPT by year of study, gender, and their interaction.

Predictor	*β* (Estimate)	SE	Wald *χ*^2^	df	*p*-value	95% CI (Lower–Upper)	AOR (e^β)
Year = Fifth × Female	0.622	0.827	0.566	1	0.452	−0.998, 2.242	1.86
Year = Fifth × Male	0.990	0.828	1.430	1	0.232	−0.633, 2.614	2.69
Year = First × Female	0.130	0.709	0.034	1	0.854	−1.260, 1.520	1.14
Year = First × Male	0.629	0.855	0.541	1	0.462	−1.046, 2.304	1.88
Year = Fourth × Female	0.680	0.789	0.742	1	0.389	−0.867, 2.227	1.97
Year = Fourth × Male	−2.127	0.968	4.831	1	0.028*	−4.023, −0.230	0.12
Year = Second × Female	−0.335	0.764	0.192	1	0.661	−1.832, 1.162	0.72
Year = Second × Male	0.413	0.854	0.234	1	0.628	−1.261, 2.088	1.51

Model summary: *χ*^2^(9) = 21.78, *p* = 0.010; Nagelkerke *R*^2^ = 0.129; Test of Parallel Lines, *p* = 0.357.

Reference group: Third-year males. Link function: Logit.

* Indicates that it is statistically significance difference.

A second ordinal logistic regression model with a cumulative logit link examined students' trust in Snapchat AI as a function of year of study, gender, and their interaction. The overall model was not statistically significant, *χ*^2^(9) = 12.17, *p* = 0.204, indicating that the inclusion of predictors did not significantly improve model fit over the null model. Goodness-of-fit statistics were acceptable [Pearson *χ*^2^(36) = 26.95, *p* = 0.86; Deviance *χ*^2^(36) = 33.65, *p* = 0.58], and the proportional-odds assumption was met (Test of Parallel Lines: *p* = 0.073). The model's explanatory power was limited (Nagelkerke *R*^2^ = 0.07). Notably, two interaction terms were statistically significant: first-year female students (*β* = −1.41, *p* = 0.044, AOR = 0.24, 95% CI: 0.06–0.96) and third-year female students (*β* = −1.48, *p* = 0.045, AOR = 0.23, 95% CI: 0.05–0.97) were significantly less likely to report higher trust in Snapchat AI compared with third-year male students. All other year-by-gender interaction terms were non-significant (Table 8).

**Table 8:** Ordinal logistic regression predicting trust in snapchat AI by year of study, gender, and their interaction.

Predictor	*β* (Estimate)	SE	Wald *χ*^2^	df	*p*-value	95% CI (Lower–Upper)	AOR (e^β)
Year = Fifth × Female	−0.957	0.807	1.407	1	0.236	−2.538, 0.624	0.38
Year = Fifth× Male	−0.069	0.800	0.007	1	0.932	−1.649, 1.511	0.93
Year = First× Female	−1.408	0.699	4.060	1	0.044*	−2.778, −0.038	0.24
Year = First × Male	−0.752	0.833	0.814	1	0.367	−2.384, 0.881	0.47
Year = Fourth × Female	−0.694	0.769	0.813	1	0.367	−2.202, 0.814	0.50
Year = Fourth × Male	−1.777	0.941	3.570	1	0.059	−3.621, 0.066	0.17
Year = Second × Female	−1.288	0.750	2.949	1	0.086	−2.757, 0.182	0.28
Year = Second × Male	−0.914	0.833	1.201	1	0.273	−2.547, 0.720	0.40
Year = Third × Female	−1.477	0.736	4.031	1	0.045*	−2.919, −0.035	0.23

Model summary: *χ*^2^(9) = 12.17, *p* = 0.204; Nagelkerke *R*^2^ = 0.073; Test of Parallel Lines, *p* = 0.073.

Reference group: Third-year males. Link function: Logit.

* Indicates that it is statistically significance difference.

## Discussion

Rapid advancements in artificial intelligence (AI) and its adoption for teaching and educational purposes could mark a new era in academia (17). Currently, the most popular AI tool among students worldwide is ChatGPT, which is considered the most rapidly expanding consumer application in history (18). In parallel, the Snapchat AI application has become available on mobile phones, particularly among the age group of undergraduate students. Metaverse has also gained significant attention in the education sector in the last two years (11). To our knowledge, this is the first survey-based study conducted among undergraduate dental students in the UAE to assess awareness, perceptions, and trust in these popular AI tools and applications.

In our study, awareness of AI applications increased across academic years, with final-year students reporting the highest confidence in their knowledge. However, ordinal logistic regression revealed that neither year of study nor gender significantly predicted awareness, suggesting that exposure alone may not determine familiarity. Interestingly, first-year students reported the highest percentage of utilization of ChatGPT (92.5%) and the highest rate of awareness of the Metaverse (32.1%), which may reflect curiosity or early adoption trends among new cohorts. Previous research indicates that students might overestimate their level of awareness about AI, and this should be taken into consideration in the future dental curriculum (19).

When it comes to using AI tools specifically as resources for studies or dental practice, fourth-year students had the highest utilization rate (41.7%). This trend may be explained by the curriculum structure at our dental college, where the fourth year primarily focuses on theoretical coursework, while the fifth year emphasizes practical and clinical training. In this study, the majority of students (78%) believed that AI has high potential in dental education. This comes in agreement with a recent study conducted in Jordan by Ajlouni et al., which showed that a majority of students (73%) agreed on the potential of ChatGPT in facilitating the learning process (20). These findings are similar to those of a study conducted in Saudi Arabia, which reported that 72% of the participants believed that AI should be incorporated into postgraduate and undergraduate dental education (19). Of the respondents, 60.7% believed that AI could be very helpful in treatment planning. Most of the results from previous studies are more or less similar to the results of the current survey (15, 21).

The primary concern among students regarding AI integration into dentistry was related to data security and patient privacy. Notably, only 31% expressed apprehension that AI might diminish the role of dental practitioners in the future, aligning with the findings of a previous survey conducted across nine Turkish dental schools (15). The findings of similar studies conducted in Saudi Arabia, but with higher concern levels reported, with almost 50%–64% agreeing that AI could replace dentists in the future (19, 22). The conclusion in the literature is that the dental field is distinct from other professions in which the human element can be replaced by algorithms. In dentistry, human traits such as accumulated experience, empathy with patients, and the trust-based relationships between patients and dentists cannot be entirely substituted by AI-operated robots (23). Different age groups of university students may have varying needs, preferences, and levels of familiarity with technological advancements.

Comparisons between ChatGPT and Snapchat AI revealed significant differences in use and perception. *post-hoc* analyses of adjusted standardized residuals indicated that ChatGPT was more often associated with “quick and effortless responses,” while Snapchat AI was more commonly reported as “not used as a source of information.” Trust was consistently higher for ChatGPT across all years. Students also expressed greater optimism about ChatGPT's role in enhancing dental education, whereas Snapchat AI was more frequently linked to neutral or negative views. These findings highlight that while both tools are accessible, students regard ChatGPT as more credible and academically relevant. In a previous multinational cross-sectional study, the majority of students intended to use ChatGPT for assignment support and anticipated that their peers would endorse its usage, implying a potential shift towards ChatGPT use becoming a standard practice among university students (24). In an exploratory study investigating the performance of ChatGPT on a wide range of dental assessments and discussing the implications for undergraduate dental education, ChatGPT provided accurate responses to the majority of knowledge-based assessments. It was found that ChatGPT has the potential to revolutionize virtual learning. The authors recommend that dental educators adapt their teaching and assessments with this tool to the benefit of the learners (25). In another study, ChatGPT was found to increase students' motivation to learn (26).

Despite the advancements of ChatGPT, it is critical to recognize its limitations (27). While it is a powerful tool for synthesizing information and enhancing learning experiences, it operates based on patterns learned from training data and lacks the ability to critically evaluate or verify information (28). This absence of inherent reliability, particularly in clinical, medical, or scientific contexts, poses a potential risk to dental students who might use it as a primary source of information. Non-evidence-based or inaccurate content can inadvertently influence their learning and decision-making processes, emphasizing the need for cautious and supplementary use alongside validated resources (29, 30). For dental educators, this underscores the importance of teaching students to critically evaluate AI-generated content and triangulate it against validated sources.

It is important to acknowledge certain limitations of this study. First, the survey was conducted at a single dental school in the UAE, which restricts the generalizability of the findings. The data may not represent the perspectives of all dental students across the country, particularly given the differences in institutional curricula, learning environments, and exposure to digital technologies. Second, the participant pool consisted predominantly of female students (70%), which introduces a potential gender bias; the results may vary if a balanced representation of genders is achieved. Although this imbalance is a limitation, it is noteworthy that similar demographic trends have been reported in previous studies, including a study conducted in the UAE, in which female students represented 68% (31), as well as in Spanish dental schools (71%) (32), India (79.5%) (33), and Saudi Arabia; this predominance likely reflects the global demographic shift toward greater female enrollment in dental education (34). Finally, the cross-sectional design limits causal inference.

Future research should aim to address these limitations by recruiting larger and more diverse samples, encompassing all dental schools in the UAE, and ensuring balanced representation of genders to explore possible differences in preferences and attitudes. Studies incorporating sufficient covariates will enable multivariable adjustment and more robust analyses. Additionally, subsequent studies should look deeper into assessing the current knowledge and attitudes towards AI tools among dental students. This will provide a richer understanding of AI's potential impact and applications of AI in dental education and practice.

## Conclusion

The rise of AI-driven technologies, such as ChatGPT, Snapchat's My AI, and virtual platforms like the Metaverse, heralds a new era in education with the potential to redefine traditional learning methods. These tools offer diverse resources and enable interactive learning experiences for the students. In the context of dental education, integrating topics on the application of AI tools into the curriculum is becoming increasingly essential. Emphasis should also be placed on fostering critical evaluation skills, ensuring that students can validate AI-provided information and align it with the principles of evidence-based dentistry.

## Data Availability

The raw data supporting the conclusions of this article will be made available by the authors, without undue reservation.
