# Supplementary material for: Are ChatGPT, My AI Snapchat, and Metaverse used by dental students as reliable sources of dental education?
Source: Front Dent Med. 2026 Jan 6;6:1673536. doi: 10.3389/fdmed.2025.1673536 (PMC12816166; doi:10.3389/fdmed.2025.1673536)
Supplement: Supplementary file 1 [file Table1.docx]

# Appendix S1. Full Questionnaire

This appendix provides the full questionnaire used in the study “Are ChatGPT, My AI Snapchat, and Metaverse used by dental students as reliable sources of dental education?”

## Section 1: Demographic Information

1. What is your gender? ( ) Male ( ) Female

2. What is your current year of study in the dental program? ( ) 1st Year ( ) 2nd Year ( ) 3rd Year ( ) 4th Year ( ) 5th Year

## Section 2: Awareness and Knowledge of AI

3. How would you rate your awareness of AI applications? 1,2,3,4, or 5 (1 = Very low, 5 = Very high)

4. Have you ever used ChatGPT? ( ) Ever used ( )Never used

5. Have you ever used Snapchat's My AI feature? ( ) Ever used ( ) Never used

6. Have you personally used Metaverse before? ( ) Ever used ( ) Never used

7. Have you personally used a grammar correction tool before? ( ) Ever used ( ) Never used

8. Have you ever used any AI applications or tools as a source of information for your studies or dental practice? ( ) Ever used ( ) Never used

## Section 3: Perceived Benefits and Challenges of AI

9. What aspects of dentistry do you think AI applications have the most potential for? (Select all that apply)

( ) Diagnosis. ( ) Treatment planning. ( ) Education. ( ) Patient communication.

( ) Patient management. ( ) Dental imaging analysis. ( ) Research and data analysis.

( ) Administrative tasks.

10. What are your primary concerns about integrating AI into dentistry? (Select all that apply.)

( ) Data security/ patient privacy. ( ) Cost of implementation. ( ) Lack of human touch.

( ) Reducing the role of the dental practitioner.

11. AI will replace human educators in the future.

( ) Strongly agree ( ) Agree ( ) Neutral ( ) Disagree ( ) Strongly disagree

12. To avoid misuse and text plagiarism, AI should be banned from universities.

( ) Strongly agree ( ) Agree ( ) Neutral ( ) Disagree ( ) Strongly disagree

13. AI applications can be integrated into practical/clinical dental education.

( ) Strongly agree ( ) Agree ( ) Neutral ( ) Disagree ( ) Strongly disagree

14. AI applications can be integrated into Theoretical dental education.

( ) Strongly agree ( ) Agree ( ) Neutral ( ) Disagree ( ) Strongly disagree

15. The AI language model can positively change how dentists communicate with patients.

( ) Strongly agree ( ) Agree ( ) Neutral ( ) Disagree ( ) Strongly disagree

16. Do you think that using AI by students for text generation should be considered as plagiarism?

( ) Strongly agree ( ) Agree ( ) Neutral ( ) Disagree ( ) Strongly disagree

17. The integration of AI in dental education will negatively affect students' creativity.

( ) Strongly agree ( ) Agree ( ) Neutral ( ) Disagree ( ) Strongly disagree

## Section 4: Use and Experience with ChatGPT

18. How frequently do you use ChatGPT?

( ) Multiple times a day ( ) Daily ( ) A few times a week ( ) Rarely ( ) Never

19. Are you using ChatGPT as a source of education or dental information?

( ) All the time ( ) Multiple times ( ) Few times ( ) Rarely ( ) Never

20. What is the main reason that makes you prefer ChatGPT as a source of information compared to other resources?

( ) Quick and effortless responses ( ) Trustable answers to my questions ( ) I never use it as a source of information

21. Are you concerned about your privacy or data security when using ChatGPT?

( ) Yes ( ) No ( ) Maybe ( ) Unapplicable

22. Do you believe that ChatGPT could enhance the overall learning experience in dental school in the future?

( ) Strongly agree ( ) Agree ( ) Neutral ( ) Disagree ( ) Strongly disagree

23. To what extent do you trust the information provided by ChatGPT for your studies?

1,2,3,4, or 5 (1 = Not at all, 5 = Complete trust)

## Section 5: Use and Experience with Snapchat’s My AI

24. How frequently do you use Snapchat’s My AI?

( ) Multiple times a day ( ) Daily ( ) A few times a week ( ) Rarely ( ) Never

25. Are you using Snapchat’s My AI as a source of education or dental information?

( ) All the time ( ) Multiple times ( ) Few times ( ) Rarely ( ) Never

26. What is the main reason that makes you prefer Snapchat’s My AI as a source of information compared to other resources?

( ) Quick and effortless responses ( ) Trustable answers to my questions ( ) I never use it as a source of information

27. Are you concerned about your privacy or data security when using Snapchat’s My AI?

( ) Yes ( ) No ( ) Maybe ( ) Unapplicable

28. Do you believe that Snapchat's AI could enhance the overall learning experience in dental school in the future?

( ) Strongly agree ( ) Agree ( ) Neutral ( ) Disagree ( ) Strongly disagree

29. To what extent do you trust the information provided by Snapchat’s My AI for your studies?

1,2,3,4, or 5 (1 = Not at all, 5 = Complete trust)
