# Supplementary material for: Are ChatGPT, My AI Snapchat, and Metaverse used by dental students as reliable sources of dental education?
Source: Front Dent Med. 2026 Jan 6;6:1673536. doi: 10.3389/fdmed.2025.1673536 (PMC12816166; doi:10.3389/fdmed.2025.1673536)
Supplement: Supplementary file 2 [file Table2.docx]

| **Comparison** | **n (Negative Ranks)** | **n (Positive Ranks)** | **Ties** | **Mean Rank (Negative)** | **Mean Rank (Positive)** | **Sum of Ranks (Negative)** | **Sum of Ranks (Positive)** | **Z value** | **p-value (2-tailed)** | **Interpretation** | **Concordance with t test** |
| --- | --- | --- | --- | --- | --- | --- | --- | --- | --- | --- | --- |
| **Trust Snapchat – Trust ChatGPT** | 103 | 12 | 53 | 59.66 | 43.79 | 6144.50 | 525.50 | -7.948 | <0.001 | Significantly higher trust in ChatGPT than Snapchat AI | Yes |

**Supplementary table:** This table summarizes the Wilcoxon signed-rank test conducted to compare paired trust scores between ChatGPT and Snapchat AI among dental students. The table presents the number of negative and positive ranks (participants who rated one tool higher than the other), the mean and sum of ranks, and the standardized Z-statistic with associated two-tailed p-value. A greater number of negative ranks indicates that more participants trusted ChatGPT over Snapchat AI. The test revealed a statistically significant difference (Z = –7.948, p < 0.001), demonstrating that ChatGPT was rated significantly more trustworthy than Snapchat AI. Results were concordant with the paired-samples t-test, confirming the robustness of findings despite non-normality.
