# Supplementary figures and images for: Are ChatGPT, My AI Snapchat, and Metaverse used by dental students as reliable sources of dental education?
Source: Front Dent Med. 2026 Jan 6;6:1673536. doi: 10.3389/fdmed.2025.1673536 (PMC12816166; doi:10.3389/fdmed.2025.1673536)

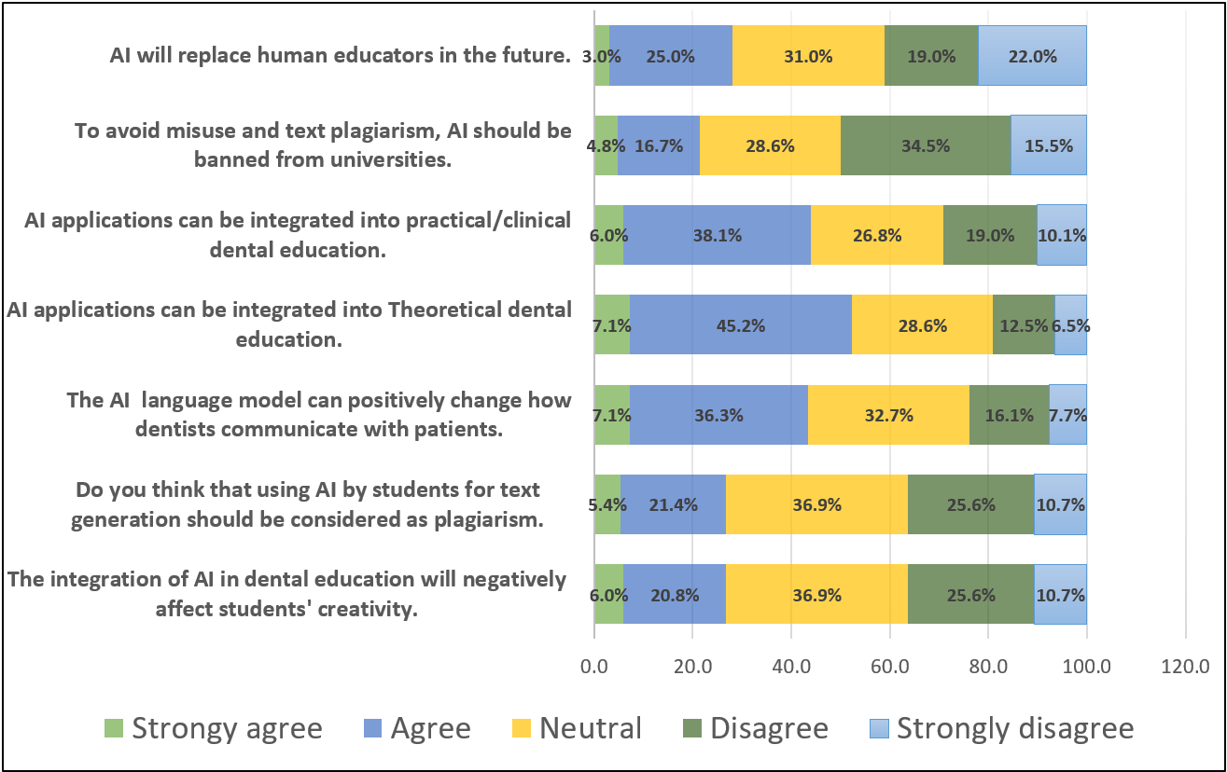

Supplement: Supplementary file 3 [file Image1.tif]
